# A personalized follow-up of kidney transplant recipients using video conferencing based on a 1-year scoring system predictive of long term graft failure (TELEGRAFT study): protocol for a randomized controlled trial

**DOI:** 10.1186/1471-2369-16-6

**Published:** 2015-01-28

**Authors:** Yohann Foucher, Aurélie Meurette, Pascal Daguin, Angélique Bonnaud-Antignac, Jean-Benoît Hardouin, Sabrina Chailan, Karine Neau, Emmanuelle Papuchon, Sandra Gaboriau, Christophe Legendre, Emmanuel Morélon, Philippe Tessier, Magali Giral

**Affiliations:** SPHERE (EA4275) - Biostatistics, Clinical Research and Pharmaco-Epidemiology, Nantes University, Nantes, France; Institut de Transplantation et de Recherche en Transplantation, ITUN, CHU Nantes, Nantes University Hospital, 30, Boulevard Jean Monnet, Nantes, 44035 France; Délégation à la recherche clinique et à l’innovation, CHU Nantes, Nantes, France; Service de Transplantation Rénale et de Soins Intensifs, Hôpital Necker, APHP and Universités Paris Descartes et Sorbonne Paris Cité, Paris, France; Service de Néphrologie, Transplantation et Immunologie Clinique, Hôpital Edouard Herriot, Lyon, France; CENTAURE Fondation, Centre d’Investigation Clinique en Biothérapie, Nantes University and INSERM U1064, Nantes, France

**Keywords:** Kidney transplantation, Personalized follow-up, Video conferencing, Randomized clinical trial

## Abstract

**Background:**

Numerous well-established clinical parameters are taken into consideration for the follow-up adaptation of kidney transplant recipients, but there are important disparities between countries, centres and clinicians. Therefore, novel scoring systems have been developed, for instance the Kidney Transplant Failure Score (KTFS) which aims to stratify patients according to their risk of return to dialysis. We hypothesize that the efficiency of the follow-up after one year post-transplantation can be improved by adapting it to the risk of graft failure defined by the KTFS estimation.

**Methods/design:**

We propose a phase IV, open label, randomized, multicentric and prospective study. The study is registered with the Clinical Trials Registry NCT01615900. 250 patients will be allocated to one of two arms: the eHealth program versus the standard of care at hospital. In the standard group, patients classified at low-risk (KTFS ≤ 4.17) will be scheduled 4 visits at hospital per year, whilst high-risk patients will visit hospital 6 times. In the eHealth group, patients classified at low-risk will be interviewed 3 times by video conferencing and once at hospital, whilst 6 visits at hospital and 6 video conferencing will be scheduled for high-risk patients.

**Discussion:**

The current study allows to scientifically evaluate the etiologic impact of a novel eHealth program. This is important to clarify the possible contribution of telemedicine in the improvement of medical follow-up. The proposed design based on 4 different sub-groups can be interesting to evaluate other personalized medicine programs.

## Background

The prevalence of renal insufficiency is increasing in all developed countries, mainly due to the ageing population, and is leading to an increased prevalence of end stage renal disease (ESRD). In France, the prevalence of ESRD was estimated to be 70,700 cases, with 56% of patients under dialysis and 44% with a functional transplant [1]. Compared to renal transplantation, extra renal dialysis is associated with higher mortality [2] and lower quality of life [3, 4]. Additionally, the overall long-term cost of transplantation is lower than that of dialysis. In France in 2007, the mean annual cost of hemodialysis for the social insurance system reached €88,608 versus €20,147 for transplantation. Quite importantly, the mean transplantation cost includes a significantly higher cost in the first year, where it is comparable to that of hemodialysis [5]. This occurs because of the higher clinical requirements immediately post transplantation, such as systematic biopsies, immunosuppressive drug adaptation, and risk of acute rejection.

Despite guidelines provided by the French health authority (HAS), the Kidney Disease Improving Global Outcomes (KDIGO) and the European Renal Best Practice (ERBP), there are important disparities in the patient follow-up between transplantation centres. Numerous well-established clinical parameters are naturally taken into consideration for years for the follow-up adaptation, but novel scoring systems have also been developed to assist physicians and clinicians for the personalization of care.

Because of this, we were prompted to develop the Kidney Transplant Failure Score (KTFS). The KTFS is based on eight clinical and biological factors collected within the first year of transplantation, that are easily measurable and non–invasive [6]. The KTFS was associated with an area under the time-dependent ROC curve of 0.78 (CI95% = [0.71, 0.86]) for a prognostic up to eight years post-transplantation. Low-risk patients (KTFS ≤ 4.17) had a 93% probability of having a functional kidney at 8 years post-transplantation. In contrast, the graft survival of the high-risk patients (KTFS > 4.17) was estimated at 70%.

In the present study, we hypothesize that the efficiency of the kidney transplant recipient follow-up after one year post-transplantation could be further improved by adapting it to the risk of graft failure defined by the KTFS estimation. We propose video conferencing in addition to the KTFS estimation at one-year in order to 1) decrease the number of visits at hospital for low-risk patients without reduction in quality of life or graft survival and 2) increase the number of visits for high-risk patients with a possible improvement in graft survival.

The primary aim of the study is to evaluate the efficiency of a personalized follow-up for kidney transplant recipients that consists of adapting the frequencies of video conferencing at home and visits at hospital depending on the KTFS value. We expect such a personalized follow-up to be cost-effective compared to the conventional in-hospital follow-up strategy for both low- and high-risk patients groups.

## Methods

### Design

**Figure 1:**
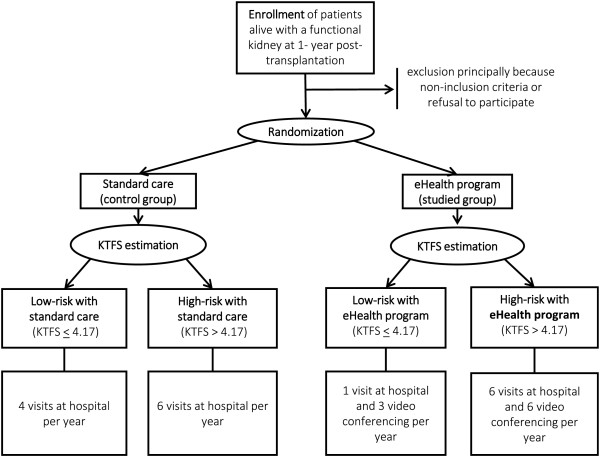
**Summary of the TELEGRAFT study design.** Circles represent the allocation process for patients into one of the four sub-groups.

This is a phase IV, open label, randomized, multicentric and prospective study. Patients are allocated to one of two arms: the novel *eHealth program* versus the *standard of care* at hospital. The 1:1 randomization of patients is stratified on centers, and performed at 1-year post-transplantation. The participation for each patient is planned for 2 years. Figure 1 outlines the study design.

#### Participants

Initial inclusion criteria were: 1) patients were alive with a functional kidney at one-year post-transplantation, 2) patients with high-speed internet access, usually digital subscriber line or fiber optic cable, 3) patients without ongoing CMV or BKV infection, 4) men or non-pregnant women, 5) patients without mental disorders and 6) patients with a written informed consent. Participants will be recruited from the University hospitals in Nantes, Paris (Necker) and Lyon (Edouard Herriot). All of these transplantation centers participate to the DIVAT and the CENTAURE networks (http://www.divat.fr, http://www.fondation-centaure.org). The recruitment will be performed at the 1-year post-transplantation hospital visit.

### Video conferencing devices

When patients are allocated in the *eHealth program*, they receive two devices: 1) A USB flash drive which allows the collection of medical information before the video conferencing. The plug-in of this device opens up a secure connection to the web via an intuitive interface specially designed for non-internet specialist patients. This hardware contains a security token for an easy and secure authentication. No software installation is necessary, only an internet connection is required, and provides web-browser independent access. 2) A tablet computer (iPad®, Apple Inc) specifically devoted for the video conferencing. Similar security is ensured by a system comparable to the USB device (token principle). Of note, the use of personal computers was first tested for 12 patients, but was concluded to be too difficult to apply in practice for several technical reasons. These included the requirement for software installation, variability in computer devices, operating systems and firewalls. Because kidney transplant recipients constitute an ageing population, we endeavored to make access as simple as possible.

### Standard care (control group)

Patients classified at low-risk of graft failure within the first 8 years post-transplantation (KTFS ≤ 4.17), will be scheduled 4 visits at hospital per year, whilst high-risk patients (KTFS > 4.17) will visit hospital 6 times. These Standard Visits (SV) consist of clinical examinations (weight and blood pressure measurements) with standard blood and urine monitoring (network file system, blood electrolytes, uremia, creatinemia and trough levels of immunosuppressive drugs). Among the 4 or 6 visits (depending of the risk group), 1 visit is devoted to a Complete Check-up (CC): detailed medical examination, additional pathology parameters (daily proteinuria, albuminemia, 25(OH)vitamin D, parathyroïd hormone, bilirubinemia, serum glutamic oxaloacetic transaminase, serum glutamic-pyruvic transaminase, Gamma-glutamyl transferase, phosphatase alkalinity, prostate specific antigen, viral serologies (Hepatitis B, Hepatitis C, HIV, CMV, EBV) and anti HLA responses screened by Luminex technology), morphologic exams (ultrasound scan and doppler sonographic of the graft artery and pulmonary X ray), and questionnaires related to quality of life (QoL) and psychological dimensions.

### eHealth program (studied group)

Patients classified at low-risk (KTFS ≤ 4.17) will be interviewed three times by video conferencing (VC). The following medical parameters are collected before the VC using the USB device: pulse, weight, temperature, and blood pressures. Only 1 CC will be performed at hospital at each anniversary of the graft (with the same complete monitoring as outlined for the control group). In contrast, six visits at hospital (1 CC and 5 SV) will be scheduled for high-risk patients (KTFS > 4.17), with six additional interposed VCs to reinforce the follow-up.

Table 1 summarizes the schedule of visits at hospital and video conferencing according to the 1-year KTFS estimation.

**Table 1:** **Schedule of the four subgroups in the TELEGRAFT study**

	High-risk (KTFS > 4.17)		Low-risk (KTFS ≤ 4.17)	
	Standard	eHealth	Standard	eHealth
Day 0 (inclusion)	CC and data collection (written consent, QoL)			
Week 03	-	VC	-	-
Week 06	SV	SV	-	-
Week 09 (Month 2)	-	VC	SV	VC
Week 12 (Month 3)	SV	SV	-	-
Week 15	-	VC	-	-
Week 18 (Month 4)	SV	SV	SV	VC
Week 21	-	VC	-	-
Week 24 (Month 6)	SV	SV	SV	VC
Week 27	-	VC	-	-
Week 30	SV	SV	-	-
Week 33	-	VC	-	-
Week 36	SV	SV	-	-
Week 39	-	VC	-	-
Week 42	SV	SV	-	-
Week 45	-	VC	-	-
Week 48	SV	SV	-	-
Week 50	-	VC	-	-
Week 52 (Month 12)	CC and data collection (pathology, QoL, morphologic exams)			
Month 13	-	VC	-	-
Month 14	SV	SV	-	-
Month 15	-	VC	SV	VC
Month 16	SV	SV	-	-
Month 17	-	VC	-	-
Month 18	SV	SV	SV	VC
Month 19	-	VC	-	-
Month 20	SV	SV	-	-
Month 21	-	VC	SV	VC
Month 22	SV	SV	-	-
Month 23	-	VC	-	-
Month 24	CC and data collection (laboratory works, QoL, morphologic screaming)			

SV, Standard Visit at hospital; CC, Complete Check-up at hospital; VC, Video Conferencing at home; Qol, Quality of Life.

### Calculation of kidney transplant failure score

The KTFS calculation is facilitated by an application available on smartphones, tablets, or computers at http://www.divat.fr/en/online-calculators. The reportable results are exported as a simple file in Portable Document Format (PDF), and contains the results in terms of patient classification and risk of return to dialysis.

### Outcome measures

The primary outcome is composite and defined by the absence of major complications until two years post-randomization, i.e. a patient is alive with a functional kidney, without acute rejection episodes, without a decrease in the graft filtration rate (eGFR) higher than 25% estimated by the Levey’s formula [7] and without cancer. Other secondary outcomes will also be analyzed to evaluate the efficiency of the eHealth program: 1) The incremental cost-effectiveness ratios (ICER) comparing eHealth with at-hospital follow-up programs, estimated from the perspective of the health care system as the mean difference in two years costs divided by the mean difference in Quality-Adjusted Life Years (QALYs) calculated using the EuroQol EQ-5D questionnaire [8]. One ICER will be estimated for both the low and high risk patient groups. 2). The evolution of the transplant-specific or generic QoL respectively measured by the ReTransQoL and the SF36 questionnaires [9–11]. 3) The evolution of other psychological dimensions related to the stress by using the ways of coping checklist [12], the anxiety/depression by using the hospital anxiety and depression scale [13] and by using the post-traumatic growth inventory [14].

All outcomes will be compared between patients allocated to the eHealth program versus those in the control standard care group. Secondarily, the same comparisons will also be performed within each subgroup of graft failure susceptibility, i.e. KTFS +/- 4.17.

### Sample size

We initially aimed to include a total of 700 patients, which was based on the number of participating centers and the workload of each center. Unfortunately, due to technical difficulties in video conferencing, we will recruit a total of ~250 transplant recipients. Assuming 90% of patients without complication in both groups for the primary outcome, a one-sided type I error level of 0.05 and a 3% non-inferiority margin, this sample size of 125 patients in each group is associated with a power at 20%. Therefore, we acknowledge that the statistical power of the TELEGRAFT study is low. 1,237 patients per group would have been necessary to reach a power of 80%. Nevertheless, a total sample size of 2,474 recipients is obviously impossible to obtain, since it represents approximately the total number of kidney transplantations for one year in France.

### Data acquisition

Demographic, clinical variables, and other specific questionnaires are collected in the DIVAT (Données Informatisées et VAlidées en Transplantation) multicentric and prospective cohort using *Integralis®,* a web-based application for data management of observational cohorts (http://www.idbc.fr)*.* The data related to the specific questionnaires are manually completed by the patients, and subsequently entered in the data base.

### Data analysis

A first descriptive analysis of the patients’ characteristics between both groups will be conducted to identify possible confounding factors, even though randomization should ensure comparability. The eHealth program versus the standard of care will be concluded as non-inferior if the lower limit of the 90% confidence interval (CI) of the difference in the primary endpoint (percentage of patients without major complications), calculated by non-parametric bootstrapping, is higher than -3%. For secondary outcomes, two-sided 95% CIs will be computed and two-tailed t-tests or chi-square statistics will be used to report p-values. 95% confidence intervals for ICER of eHealth versus at-hospital follow-up programs will be estimated using non-parametric bootstrapping. Results of the cost-effectiveness will also be analyzed using acceptability curves, by plotting the probability of eHealth program compared to standard of care follow up being cost-effective against the willingness to pay for a QALY. Deterministic sensitivity analyses will also be performed to assess the robustness of results depending on the cost of the video conferencing system. The evolution over time of each psychological dimension will be modeled using latent mixed regression Rasch models [15]. These models allow the responses to the items of each dimension to be transformed in a quantitative measure that represents the concept studied by this dimension, and allows measures to be explained by covariates. These models take into account the repeatability of the measures for each patient by using a random effect. For the dimensions where the Rasch model will not fit the data, the score computed as the sum of the items of this dimension will be modeled using a mixed linear model. With these models, the effect of the eHealth program on the evolution of the psychological measures will be determined, and this effect will be adjusted on other covariates (sex, age, risk classification…).

### Ethical approval and registration

The study is registered with the Clinical Trials Registry (NCT01615900) and has approval from the ethical Committee for Persons’ Protection (CPP, Tours, 2011-R30).

### Schedule

After initial refinement, in particular the resolution of technical issues related to the video conferencing by choosing a tablet computer, only the Nantes University hospital began the study with an inclusion of 38 patients. Patient inclusions from the other centers are possible from September 2014. Based on the yearly number of transplantations performed among the three participating centers, we expect to include 250 patients by September 2016. In line with the two-year follow up, the end of data collection is anticipated by September 2018, and publication of the results in 2019.

## Discussion

The cost-effectiveness of renal transplantation for ESRD patients is well-established compared to long-term dialysis [16–18]. Nevertheless, because the follow-up of the kidney transplant recipient is often performed at hospitals several times per year, is often coupled with significant travel requirements, long queue times and the stress associated with medical examinations, this places a measureable increase in costs and may impact the recipient’s QoL. This issue is even more pertinent when considering the increased prevalence of patients living with a functional kidney; 31,000 French patients in 2011 [1]. We hypothesized that video conferencing at home by using USB device and tablet computer may improve the efficiency of kidney transplant recipient follow-up. Such web- or mobile-based communication systems have already been considered as possible improvements for medical follow-up for other diseases; for instance in diabetes patients [19], in women after gynecological surgery [20], or for patients with chronic obstructive pulmonary disease [21].

One very important and relevant potential benefit of our adaptation of the video conferencing frequency is in regard to the frailty of patients, i.e. their risk of graft failure evaluated by the KTFS [6]. The aim of the TELEGRAFT study is to determine whether video conferencing could be used to individualize patients’ care so as to lighten (reinforce) follow-up in low (high) risk patients as compared to standard of care at hospital. This reallocation of health care resources could be profitable in many aspects. On one hand, it might increase the QoL of low-risk patients by offering them living conditions closer to those of the general healthy population whilst lowering their medical follow-up. On the other hand, it might improve the management of the daily queue at hospitals thus allowing physicians to allocate more time to high-risk patients. Our study will propose to evaluate these different dimensions and also to investigate adherence of patients and physicians.

Even if the randomized study we propose suffers from a small statistical power, the analysis will be based on a control group, i.e. comparable patients following standard of care. This design allows etiologic results without confounders, as requested by the Committee on Evaluating Clinical Applications of Telemedicine [22], in contrast to the majority of the literature in Telemedicine which is based on observational studies [23–25]. This may explain why the possible contribution of telemedicine in the improvement of medical follow-up is currently not clear [26, 27].

In conclusion, the current study allows to scientifically evaluate the etiologic impact of a novel eHealth program for the medical follow-up of kidney transplant recipients. We believe that the results will have an important impact in the transplantation community. Additionally, because the medical care of people with chronic disease is under the spotlight given the growing prevalence of such conditions in ageing populations, the proposed randomized design with 4 different sub-groups can be interesting to evaluate other personalized medicine programs.

## Abbreviations

KTR: Kidney transplant recipient
KTFS: Kidney transplant failure score
ESRD: End stage renal disease
HAS: French health authority
KDIGO: Kidney disease improving global outcomes
ERBP: European renal best practice
ROC: Receiver operating characteristic
CMV: CytoMegaloVirus
BKV: BK virus
CC: Complete check-up
HIV: Human immunodeficiency virus
EBV: Epstein-barr virus
QoL: Quality of life
VC: Video conferencing
DIVAT: Données informatisées et validées en transplantation
ICER: Incremental cost-effectiveness ratio
CI: Confidence interval.
